# Supplementary material for: Key homeobox transcription factors regulate the development of the firefly’s adult light organ and bioluminescence
Source: Nat Commun. 2024 Mar 5;15:1736. doi: 10.1038/s41467-024-45559-7 (PMC10914744; doi:10.1038/s41467-024-45559-7)
Supplement: Supplementary file 1 — Supplementary Information [file 41467_2024_45559_MOESM1_ESM.pdf]

Supplementary Information

Key homeobox transcription factors regulate the development of the firefly's adult light organ and bioluminescence

Xinhua Fu and Xinlei Zhu

Contents

Supplementary Fig. 1 3
Supplementary Fig. 2 4
Supplementary Fig. 3 5
Supplementary Fig. 4 6
Supplementary Fig. 5 7
Supplementary Fig. 6 9
Supplementary Fig. 7 10
Supplementary Fig. 8 11
Supplementary Fig. 9 12
Supplementary Fig. 10 13
Supplementary Fig. 11 14
Supplementary Fig. 12 15
Supplementary Fig. 13 16
Supplementary Fig. 14 17
Supplementary Fig. 15 18
Supplementary Fig. 16 19
Supplementary Table 1 20
Supplementary Table 2 21
Supplementary Table 3 22
Supplementary Table 4 23
Supplementary Table 5 24
Supplementary Table 6 25
Supplementary Table 7 26
Supplementary Table 8 27
Supplementary Table 9 28
References 29

Supplementary Figures

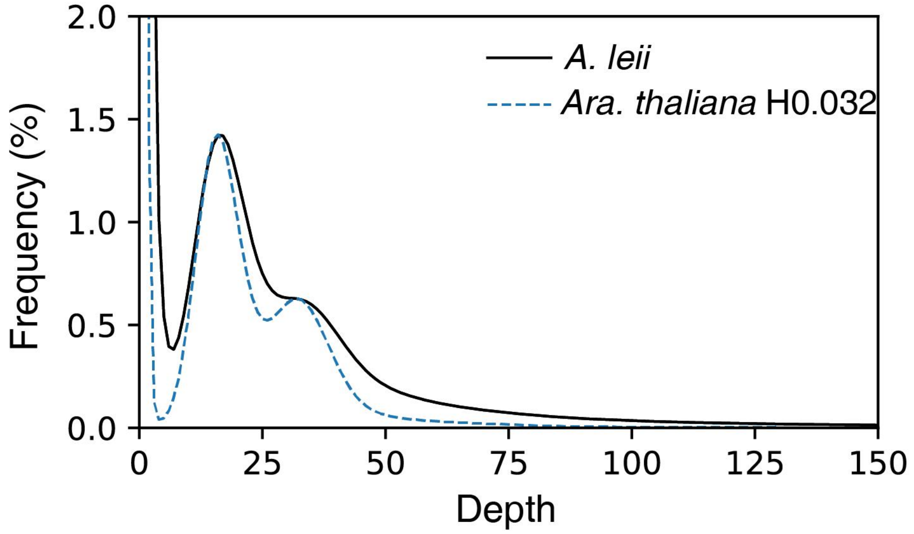

Supplementary Fig. 1. An estimate of the *A. leii* genome size with k-mer. Heterozygosity and repeat content of the *A. leii* genome were estimated by combining the simulation data results of *Arabidopsis* with 3.2% heterozygosity and the frequency peak distribution of k-mer. Genome Size =1,094,479,203 bp, genome heterozygosity=3.20%.

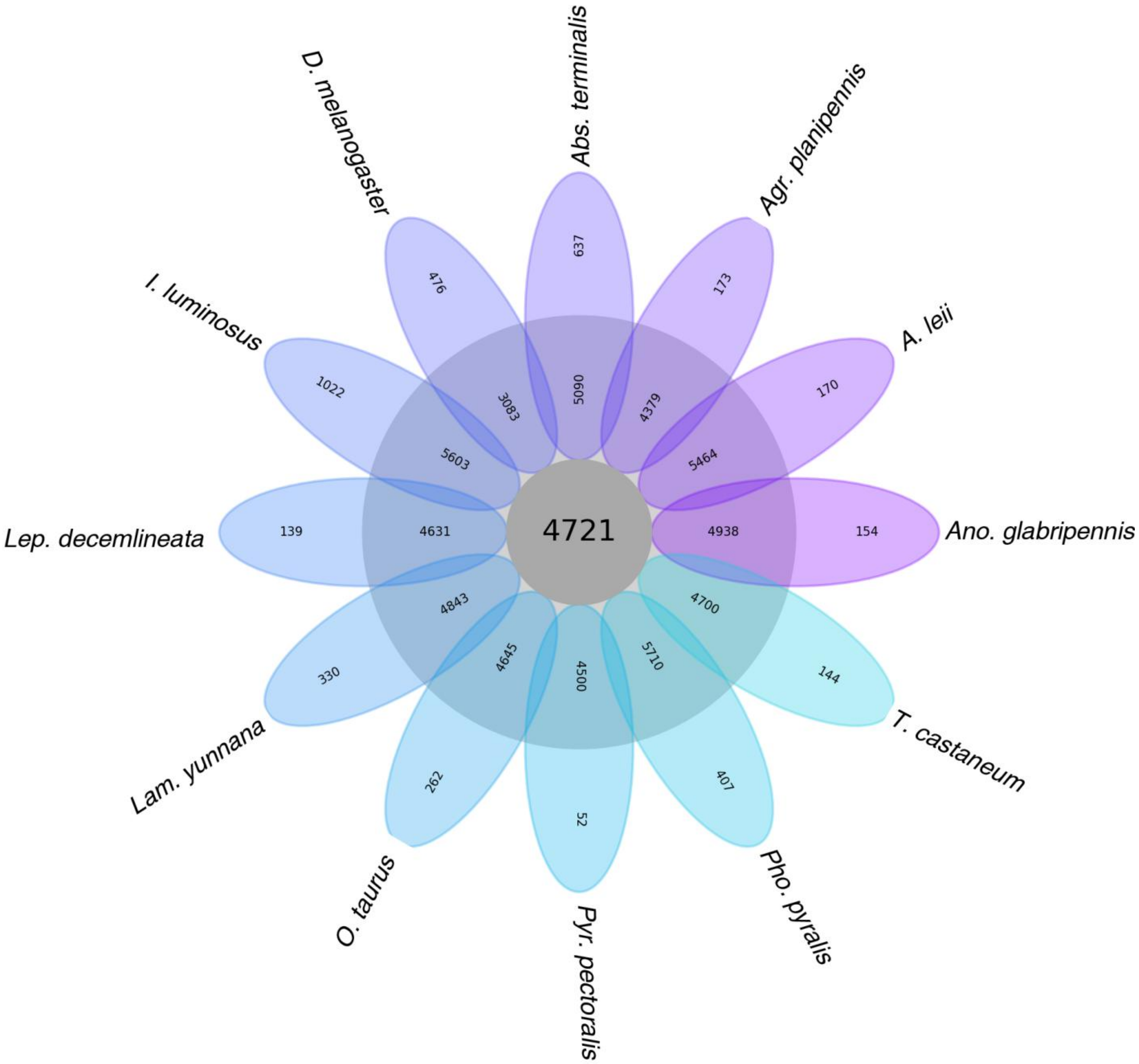

Supplementary Fig. 2. Clusters of Orthologous Groups (COG) annotation of the core genes in 12 insect genomes. A total of 4721 single-copy orthogroups were shared among the 12 species. The inner circle and the outer circle represent the putative orthogroups and the unigenes, respectively, in the 12 insect genomes. Source data are provided as a Source Data file.

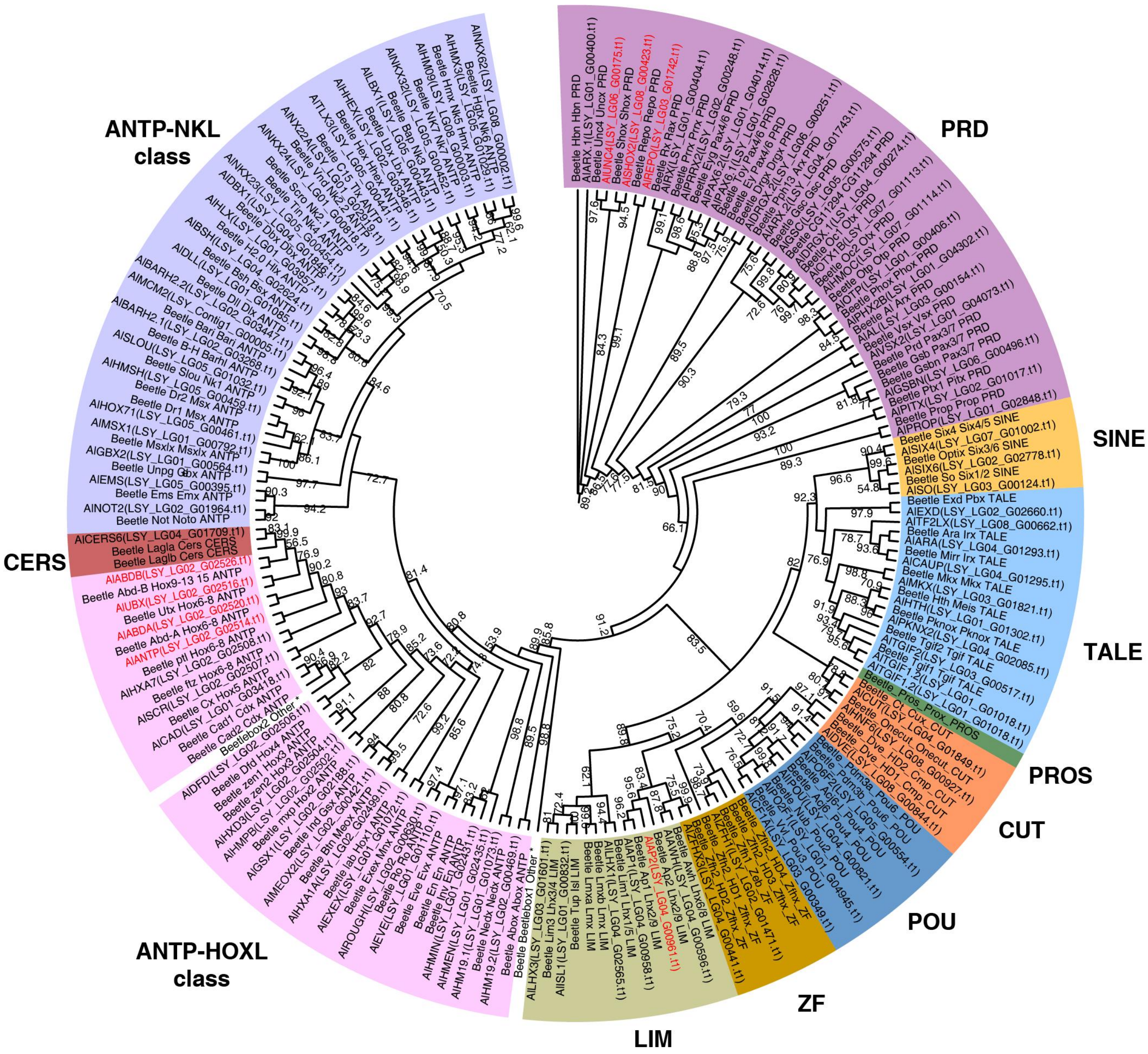

Supplementary Fig. 3. Evolutionary relationships of homeobox genes in *A. leii* and *T. castaneum*. Homeobox genes of *A. leii* are named by gene (genome ID) and homeobox Genes of *T. castaneum* are named by beetle+gene+class+family. Families are labeled using colored shading. Genes with functional verification are highlighted. Other types of homeobox genes are highlighted using a star (*) symbol.

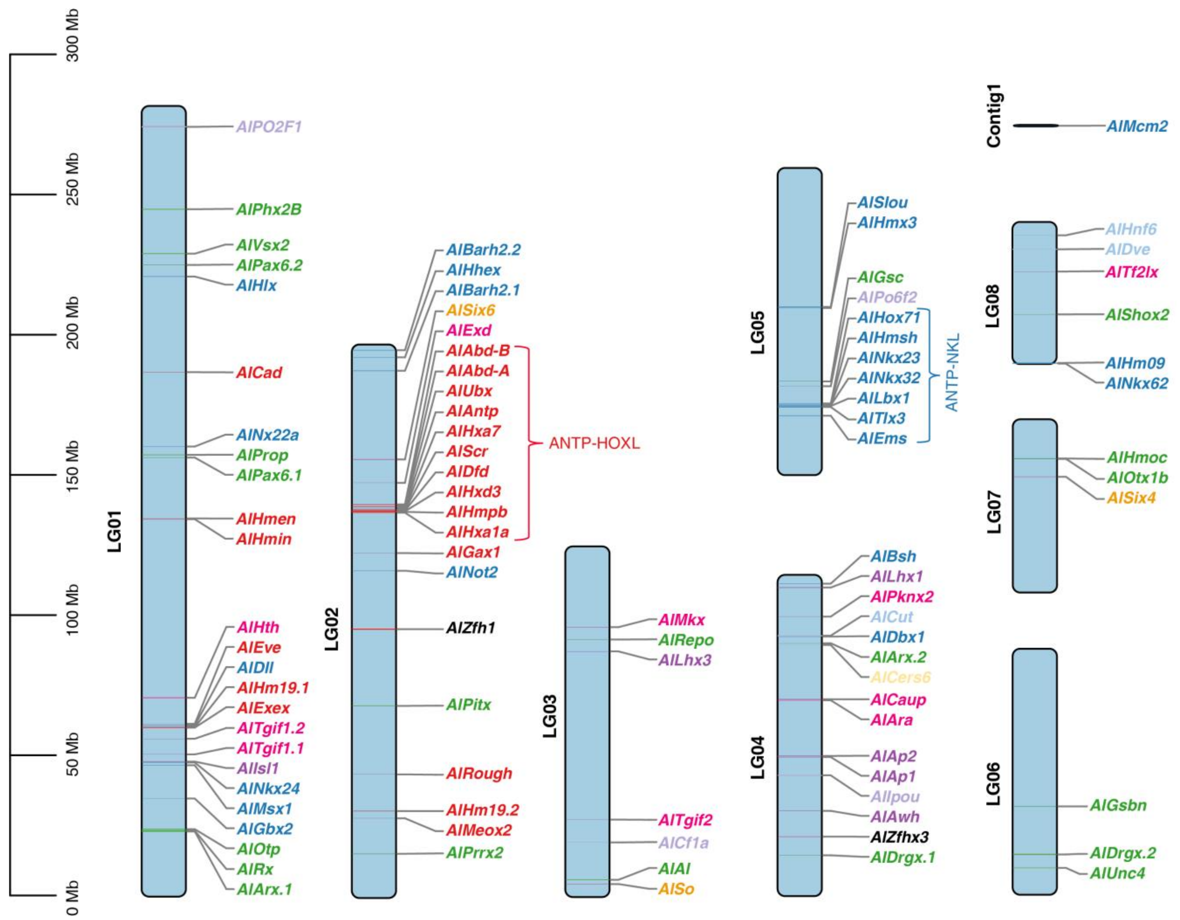

Supplementary Fig. 4. Distribution of homeobox genes on *A. leii* chromosomes. HOXL class family of genes is shown in red, NKL class - blue, PRD family - green, LIM family - purple, POU family - pale purple (lilac), SINE family - orange, TALE family - pink, CUT family - pale blue, ZF family - black, and CERS family - yellow.

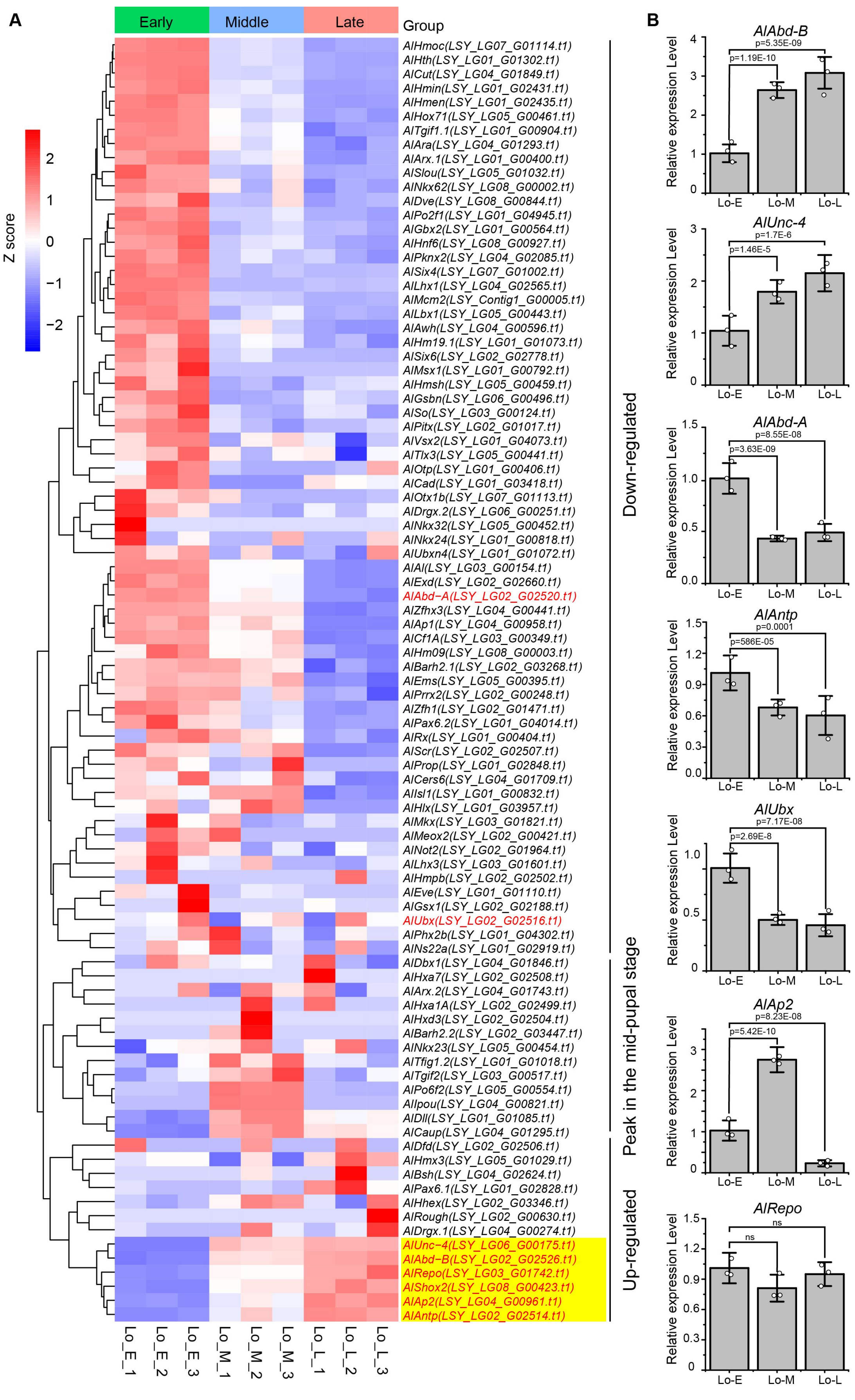
A
Early
Middle
Late
Group
Z score
2
1
0
−1
−2
AlHmoc(LSY_LG07_G01114.t1)
AlHth(LSY_LG01_G01302.t1)
AlCut(LSY_LG04_G01849.t1)
AlHmin(LSY_LG01_G02431.t1)
AlHmen(LSY_LG01_G02435.t1)
AlHox71(LSY_LG05_G00461.t1)
AlTgif1.1(LSY_LG01_G00904.t1)
AlAra(LSY_LG04_G01293.t1)
AlArx.1(LSY_LG01_G00400.t1)
AlSlou(LSY_LG05_G01032.t1)
AlNkx62(LSY_LG08_G00002.t1)
AlDve(LSY_LG08_G00844.t1)
AlPo2f1(LSY_LG01_G04945.t1)
AlGbx2(LSY_LG01_G00564.t1)
AlHnf6(LSY_LG08_G00927.t1)
AlPknx2(LSY_LG04_G02085.t1)
AlSix4(LSY_LG07_G01002.t1)
AlLhx1(LSY_LG04_G02565.t1)
AlMcm2(LSY_Contig1_G00005.t1)
AlLbx1(LSY_LG05_G00443.t1)
AlAwh(LSY_LG04_G00596.t1)
AlHm19.1(LSY_LG01_G01073.t1)
AlSix6(LSY_LG02_G02778.t1)
AlMsx1(LSY_LG01_G00792.t1)
AlHmsh(LSY_LG05_G00459.t1)
AlGsbn(LSY_LG06_G00496.t1)
AlSo(LSY_LG03_G00124.t1)
AlPitx(LSY_LG02_G01017.t1)
AlVsx2(LSY_LG01_G04073.t1)
AlTlx3(LSY_LG05_G00441.t1)
AlOtp(LSY_LG01_G00406.t1)
AlCad(LSY_LG01_G03418.t1)
AlOtx1b(LSY_LG07_G01113.t1)
AlDrgx.2(LSY_LG06_G00251.t1)
AlNkx32(LSY_LG05_G00452.t1)
AlNkx24(LSY_LG01_G00818.t1)
AlUbxn4(LSY_LG01_G01072.t1)
AlAl(LSY_LG03_G00154.t1)
AlExd(LSY_LG02_G02660.t1)
AlAbd−A(LSY_LG02_G02520.t1)
AlZfhx3(LSY_LG04_G00441.t1)
AlAp1(LSY_LG04_G00958.t1)
AlCf1A(LSY_LG03_G00349.t1)
AlHm09(LSY_LG08_G00003.t1)
AlBarh2.1(LSY_LG02_G03268.t1)
AlEms(LSY_LG05_G00395.t1)
AlPrrx2(LSY_LG02_G00248.t1)
AlZfh1(LSY_LG02_G01471.t1)
AlPax6.2(LSY_LG01_G04014.t1)
AlRx(LSY_LG01_G00404.t1)
AlScr(LSY_LG02_G02507.t1)
AlProp(LSY_LG01_G02848.t1)
AlCers6(LSY_LG04_G01709.t1)
AlIsl1(LSY_LG01_G00832.t1)
AlHlx(LSY_LG01_G03957.t1)
AlMkx(LSY_LG03_G01821.t1)
AlMeox2(LSY_LG02_G00421.t1)
AlNot2(LSY_LG02_G01964.t1)
AlLhx3(LSY_LG03_G01601.t1)
AlHmpb(LSY_LG02_G02502.t1)
AlEve(LSY_LG01_G01110.t1)
AlGsx1(LSY_LG02_G02188.t1)
AlUbx(LSY_LG02_G02516.t1)
AlPhx2b(LSY_LG01_G04302.t1)
AlNs22a(LSY_LG01_G02919.t1)
AlDbx1(LSY_LG04_G01846.t1)
AlHxa7(LSY_LG02_G02508.t1)
AlArx.2(LSY_LG04_G01743.t1)
AlHxa1A(LSY_LG02_G02499.t1)
AlHxd3(LSY_LG02_G02504.t1)
AlBarh2.2(LSY_LG02_G03447.t1)
AlNkx23(LSY_LG05_G00454.t1)
AlTfig1.2(LSY_LG01_G01018.t1)
AlTgif2(LSY_LG03_G00517.t1)
AlPo6f2(LSY_LG05_G00554.t1)
AlIpou(LSY_LG04_G00821.t1)
AlDll(LSY_LG01_G01085.t1)
AlCaup(LSY_LG04_G01295.t1)
AlDfd(LSY_LG02_G02506.t1)
AlHmx3(LSY_LG05_G01029.t1)
AlBsh(LSY_LG04_G02624.t1)
AlPax6.1(LSY_LG01_G02828.t1)
AlHhex(LSY_LG02_G03346.t1)
AlRough(LSY_LG02_G00630.t1)
AlDrgx.1(LSY_LG04_G00274.t1)
AlUnc−4(LSY_LG06_G00175.t1)
AlAbd−B(LSY_LG02_G02526.t1)
AlRepo(LSY_LG03_G01742.t1)
AlShox2(LSY_LG08_G00423.t1)
AlAp2(LSY_LG04_G00961.t1)
AlAntp(LSY_LG02_G02514.t1)
Down-regulated
Peak in the mid-pupal stage
Up-regulated
Lo_E_1
Lo_E_2
Lo_E_3
Lo_M_1
Lo_M_2
Lo_M_3
Lo_L_1
Lo_L_2
Lo_L_3
B
AlAbd-B
p=5.35E-09
p=1.19E-10
Relative expression Level
Lo-E
Lo-M
Lo-L
AlUnc-4
p=1.7E-6
p=1.46E-5
AlAbd-A
p=8.55E-08
p=3.63E-09
AlAntp
p=0.0001
p=586E-05
AlUbx
p=7.17E-08
p=2.69E-8
AlAp2
p=8.23E-08
p=5.42E-10
AlRepo
ns
ns

Supplementary Fig. 5. Expression levels of homeobox genes in different development stages of ventrites 6 and 7, corresponding to the adult light organs. (A) Heatmap shows the expression levels of homeobox genes in different development stages of male adult light organs by RNA-seq, n=3 Biological duplication. Lo, male adult light organs; E, early pupal stage; M, middle pupal stage; L, late pupal stage. Up-regulated homeobox genes are shown in yellow. The genes that were functionally verified in subsequent experiments are marked in red. Data were z-score normalized within a given parameter, and organized by hierarchical clustering. The expression patterns of these homeobox transcription factors can be broadly categorized into three types: down-regulated during the development of the light organ, peak expression in the mid-pupal stage, and down-regulated during the development of the male light organ. (B) The quantitative RT-PCR results showing the expression levels of *AlAbd-A, AlAbd-B, AlUbx, AlAntp*, *AlUnc-4*, *AlRepo* and *AlAp2* in different development stages of female adult light organs. Data shown are the mean value ± SD from 3 biological replicates. The statistical significance was derived using two-sided student's t-tests, where ns stands for nonsignificant ($p>0.05$). Source data are provided as a Source Data file.

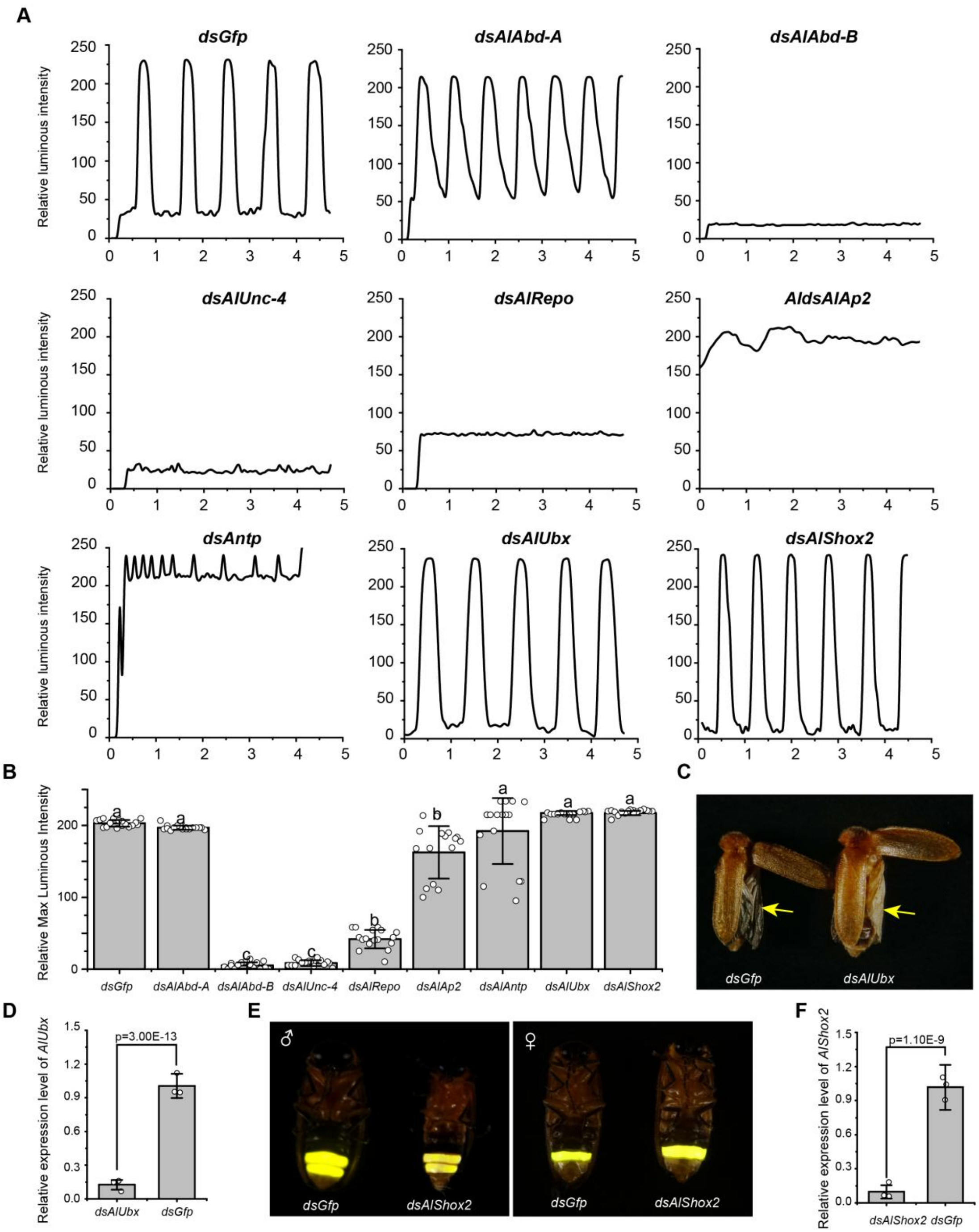

Supplementary Fig. 6. Functional verification of homeobox genes. (**A**) Flash patterns of the adults in Supplementary Movie 1. (**B**) Comparisons of relative maximum light intensity between different RNAi treatments (n=16 samples). Different lowercase letters above bars indicate significant differences as determined by one-way ANOVA and Tukey's HSD test ($p < 0.05$). (**C**) and (**E**) The phenotypes of *dsAlUbx* (**C**) and *dsAlShox2* (**E**). The yellow arrowheads indicate hindwings. *dsAlUbx* treatment resulted in pale yellow hindwings. (**D**) and (**F**) The fluorescence quantitative PCR analysis of RNAi of *dsAlUbx* (**D**) and *dsAlShox2* (**F**) compared to the *dsGfp* control. The data represent the average ± SD of three biological replicates (2 males and 1 female). The statistical significance was derived using two-sided student's t-tests**.** Source data are provided as a Source Data file.

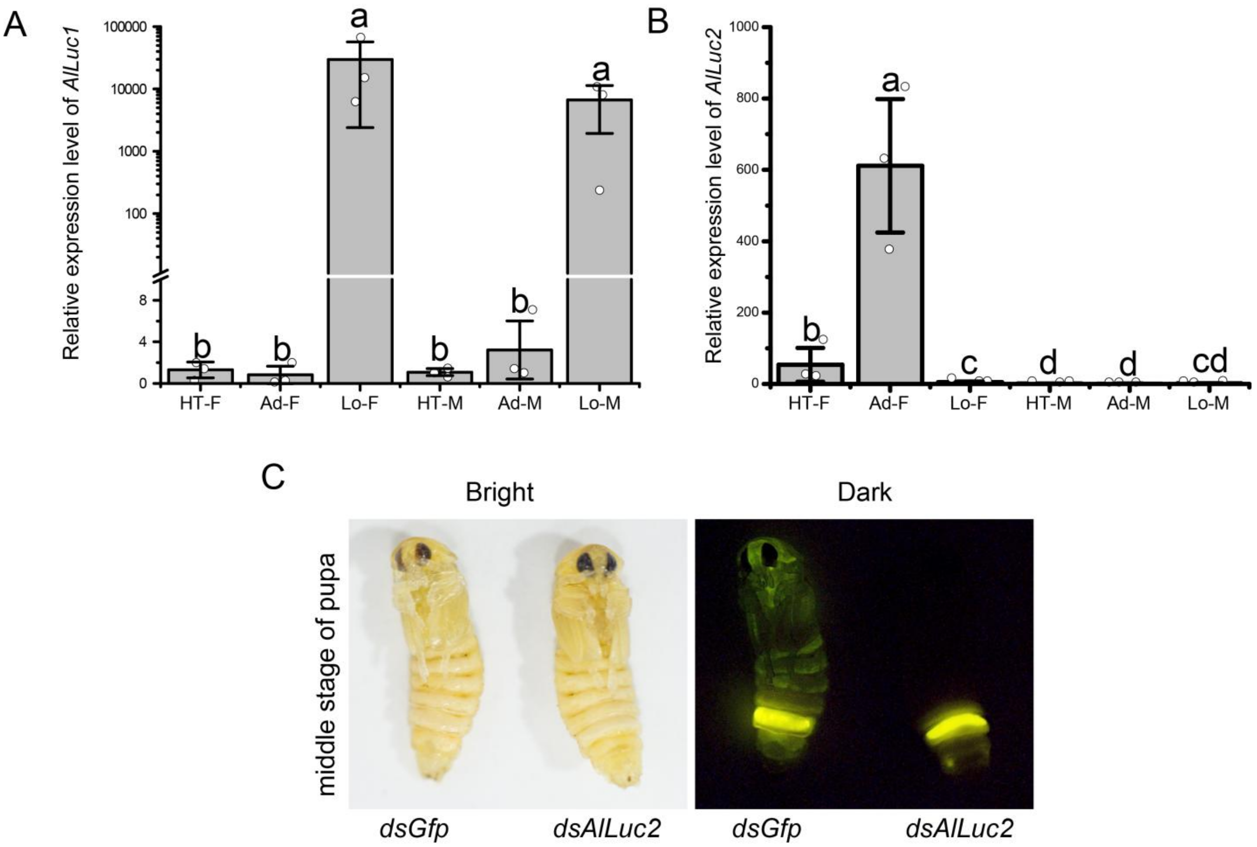

Supplementary Fig. 7. Expression patterns of *AlLuc1* and *AlLuc2* in *A. leii*. (A) and (B) Expression of *AlLuc1* (A) and *AlLuc2* (B) in different tissues of adults by qPCR. The data are presented as mean values ± SD of three biological replicates. F, female; M, male; HT, head and thorax; Lo, adult light organs; Ad, abdominal tissues without light organs. Different lowercase letters above bars indicate significant differences as determined by the Fisher test ($p < 0.05$). Log10 scale after break on Y axis. **(C)** The phenotype of *AlLuc2* knockdown in female pupae in dark and bright conditions. Adult light organs functioned normally in *dsAlLuc2* female pupae but head, thorax, and first five abdominal segments were not luminescent. Source data are provided as a Source Data file.

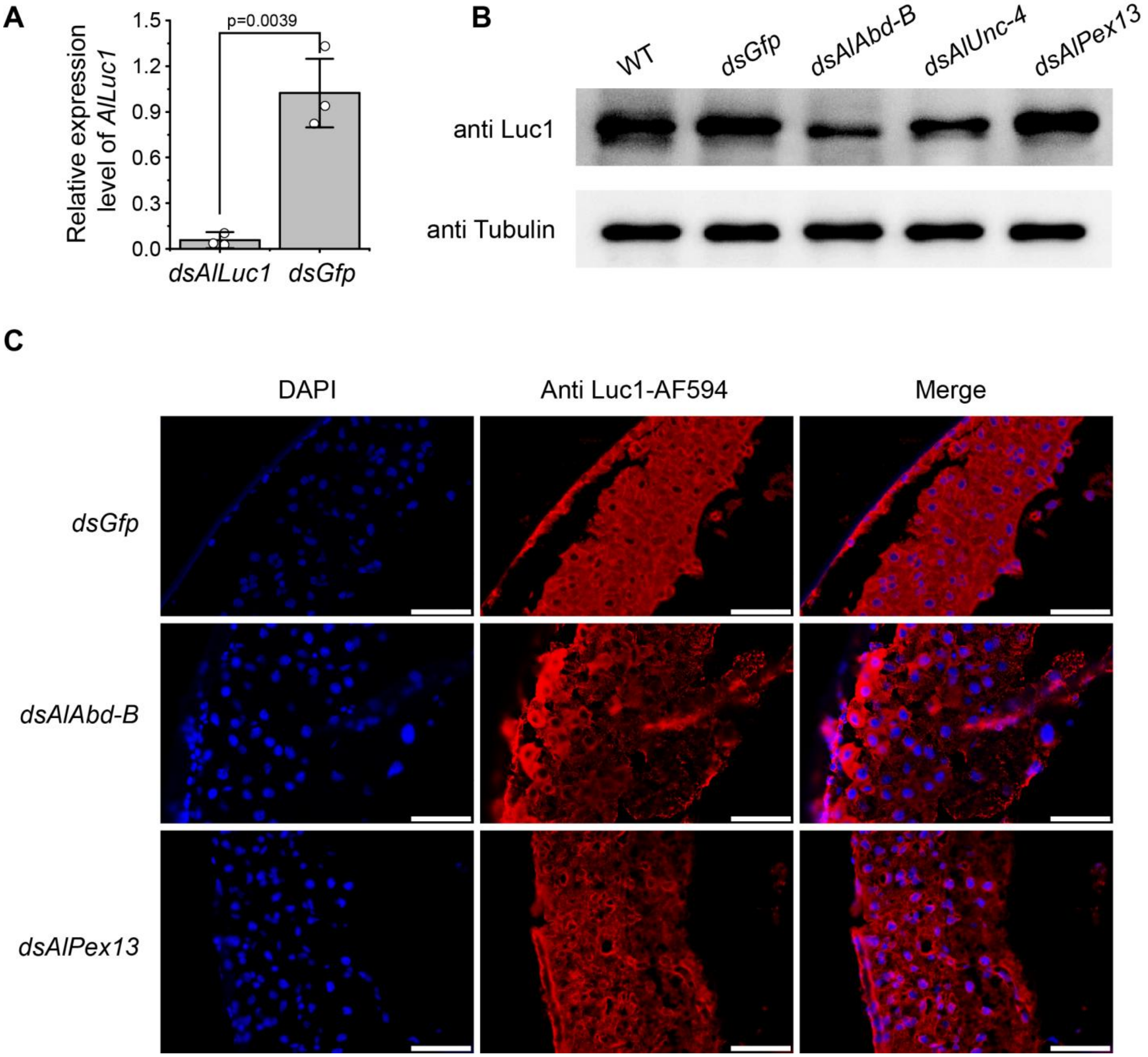

Supplementary Fig. 8. Expression level of *AlLuc1*. (A) The qPCR result of *dsAlLuc1* in three replicates compared to the control (ds*Gfp*). The data are presented as mean values ± SD of three biological replicates (2 males and 1 female). The significance was derived using two-sided student's t-tests. **(B)** AlLuc1 protein expression levels were detected by immunoblots with antibodies of Luc1 antibody in wild type (WT), *dsGfp*, *dsAlAbd-B*, *dsUnc-4*, and *dsPex13* groups (3 individuals in each group). Anti-tubulin antibody was used as control loading. n=2 independent experiments. **(C)** Immunofluorescence staining of localization anti-Luc1-AF594 antibodies in the photogenic layer of *dsAlAbd-B* and *dsAlPex13*, n=3 independent experiments. Scale bar = 50 μm, DAPI was used to stain the nucleus. Source data are provided as a Source Data file.

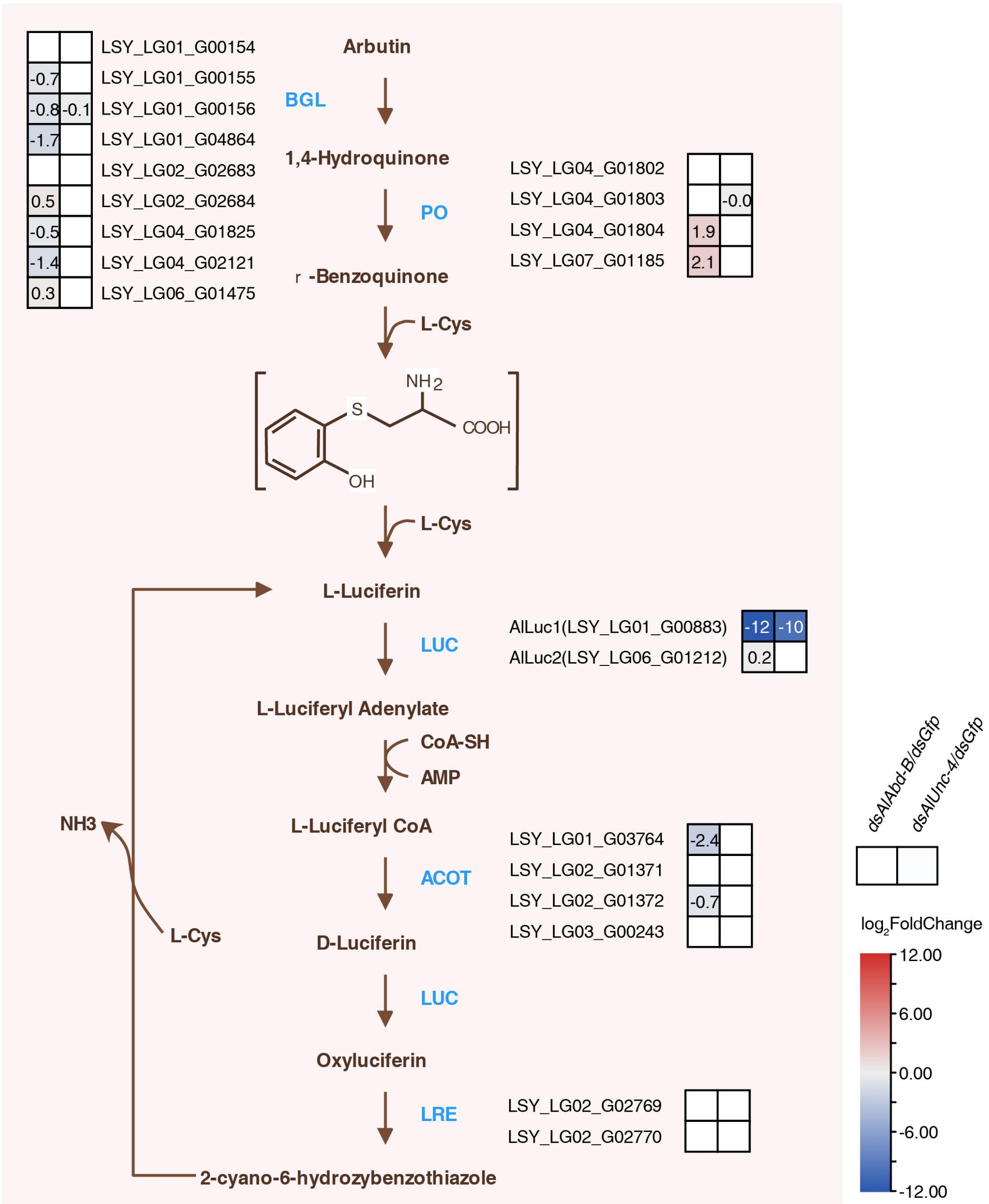

Supplementary Fig. 9. Expression profiles of the luciferin biosynthesis pathway candidate genes in *AlAbd-B* RNAi and *AlUnc-4* RNAi. Heatmap visualization of relative mRNA abundance was based on FPKM by RNA-seq. Relative transcript abundance was normalized using *dsGfp* as the control and log2-transformed. Gene expression levels are indicated using a color scale from blue (down-regulated) to red (up-regulated), with $\log_2$FoldChange marked in blocks. White boxes correspond to genes without differential expression compared to the *dsGfp* control. Data represent means of three biological replicates. The pathway adapted from [1-3]. Source data are provided as a Source Data file.

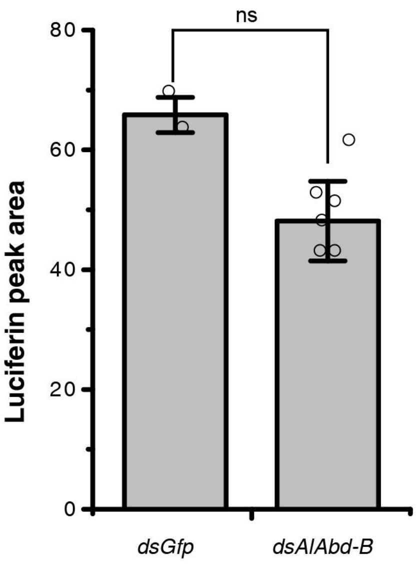

Supplementary Fig. 10. Comparisons of luciferin content between ds*Gfp* and *dsAlAbd-B* by HPLC. The data are presented as mean values ± SD, *dsGfp* group n=2 males samples, *dsAlAbd-B* group n=6 males samples. The two groups were compared using one-way ANOVA and Tukey's multiple range test, where ns stands for nonsignificant (p>0.01). Source data are provided as a Source Data file.

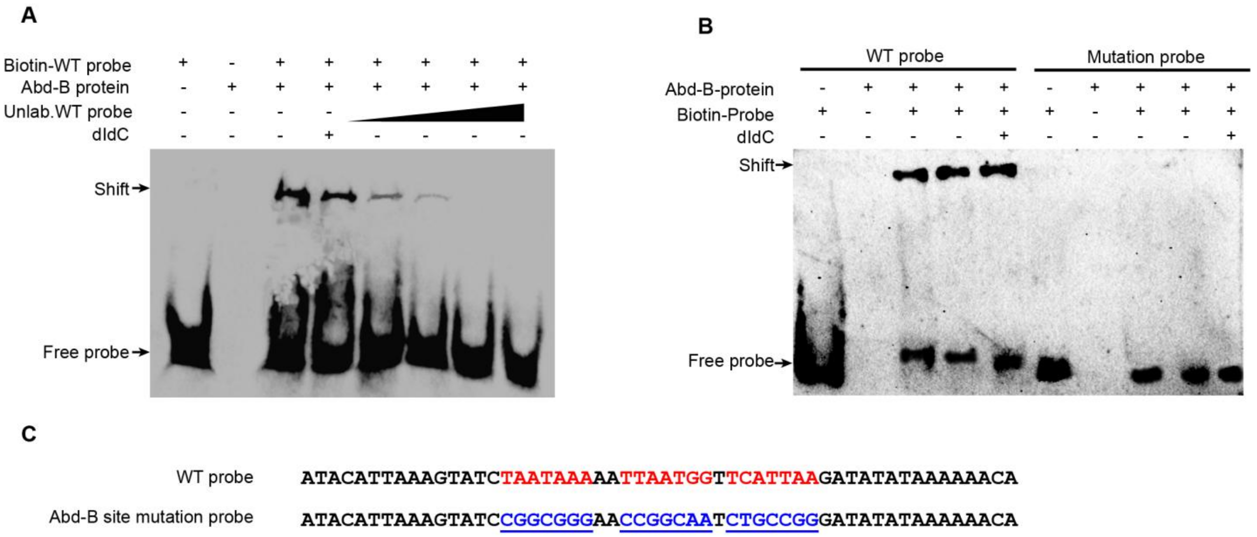

Supplementary Fig. 11. Electrophoretic mobility shift assay (EMSA) of AlABD-B and *AlLuc1* promoter. (**A**) and (**B**) Binding of AlABD-B to the promoter of *AlLuc1* in EMSA. The biotin-labeled probes were incubated with purified pET32a-AlAbd-B fusion protein to detect the binding ability, and the unlabeled (A) or mutant probe (B) was used as a competitor to test the binding specificity of AlABD-B. n=2 independent experiments. (**C**) Oligonucleotide probes used for EMSA. The WT probe corresponds to ABD-B regulatory elements (red) in the *AlLuc1* promoter. The mutant probe was the same as the wild-type probe, except for T being substituted with C, and A with G (blue) .

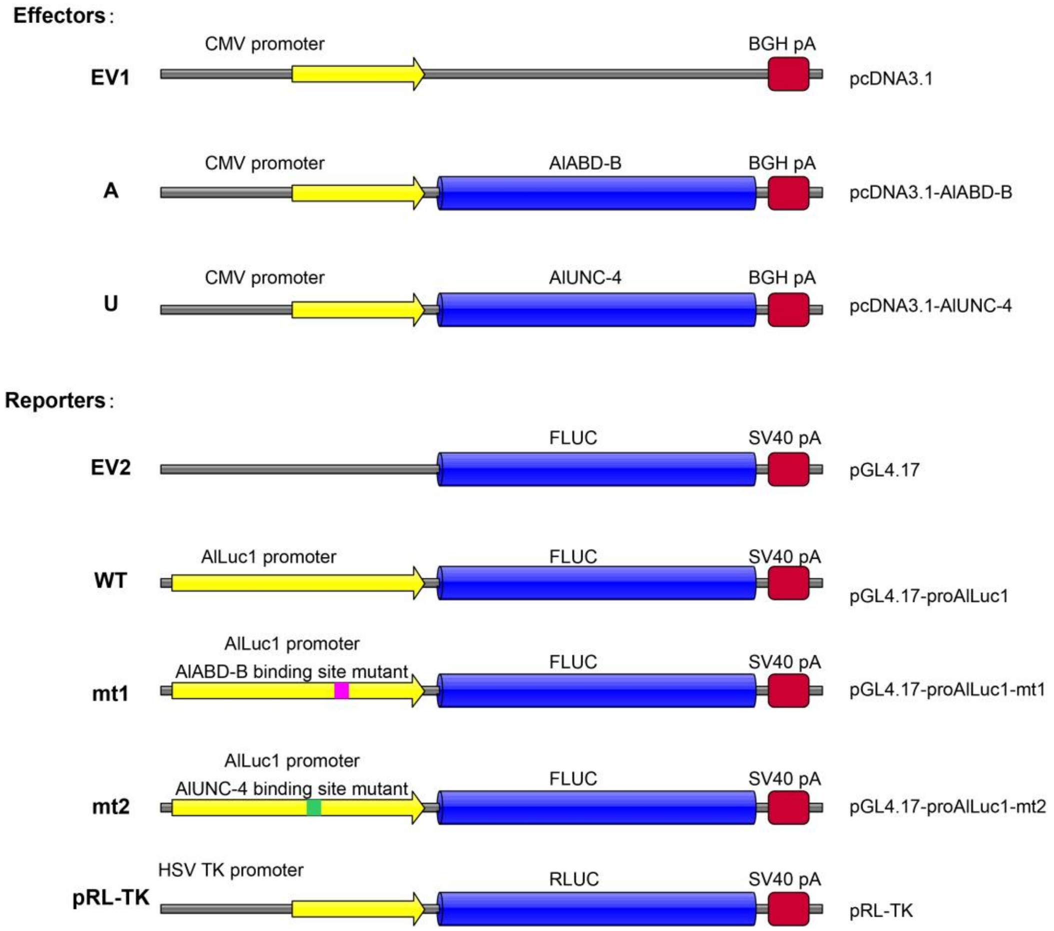

Supplementary Fig. 12. Vector constructs used in dual-luciferase assay. Top, maps of the effector plasmids: *AlAbd-B* (A) and *AlUnc-4* (U) were cloned into a pcDNA3.1 expression vector (EV1), driven by the CMV promoter. Bottom, maps of the reporter plasmids: *proAlLuc1*(WT) was cloned into a pGL 4.17 expression vector (EV2). The AlABD-B (purple square) and AlUNC-4 binding sits (green square) were mutated individually on proAlLuc1, and designated as pGL 4.17-proAlLuc1-mt1 (mt1) and pGL 4.17-proAlLuc1-mt2 (mt 2). pRL-TK corresponding to the renilla luciferase reporter, and driven by the HSV-TK promoter, was used as a control for the transfection efficiency.

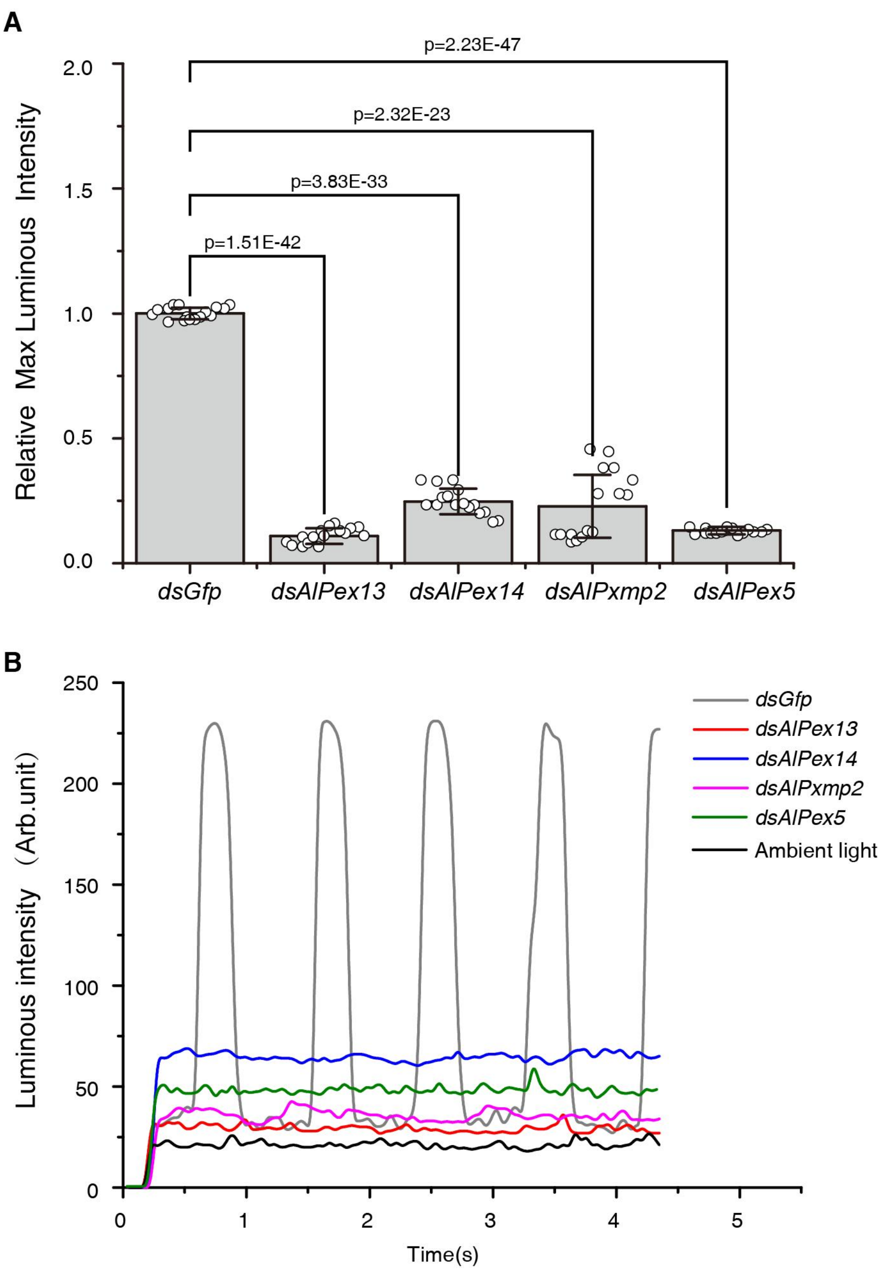

Supplementary Fig. 13. Flash patterns of the *dsGfp*, *dsAlPex13*, *dsAlPex14*, *dsAlPex5* and *dsAlPxmp2* groups. **(A) C**omparisons of the maximum relative flash light intensity among Pexs RNAi groups. All data are shown as the mean value of 16 replicates. Error bars represent SD. The data was analyzed using one-way ANOVA and Tukey's multiple range tests, with ds*Gfp* as the control. **(B)** Flash patterns of adults in Supplementary Movie 3. Source data are provided as a Source Data file.

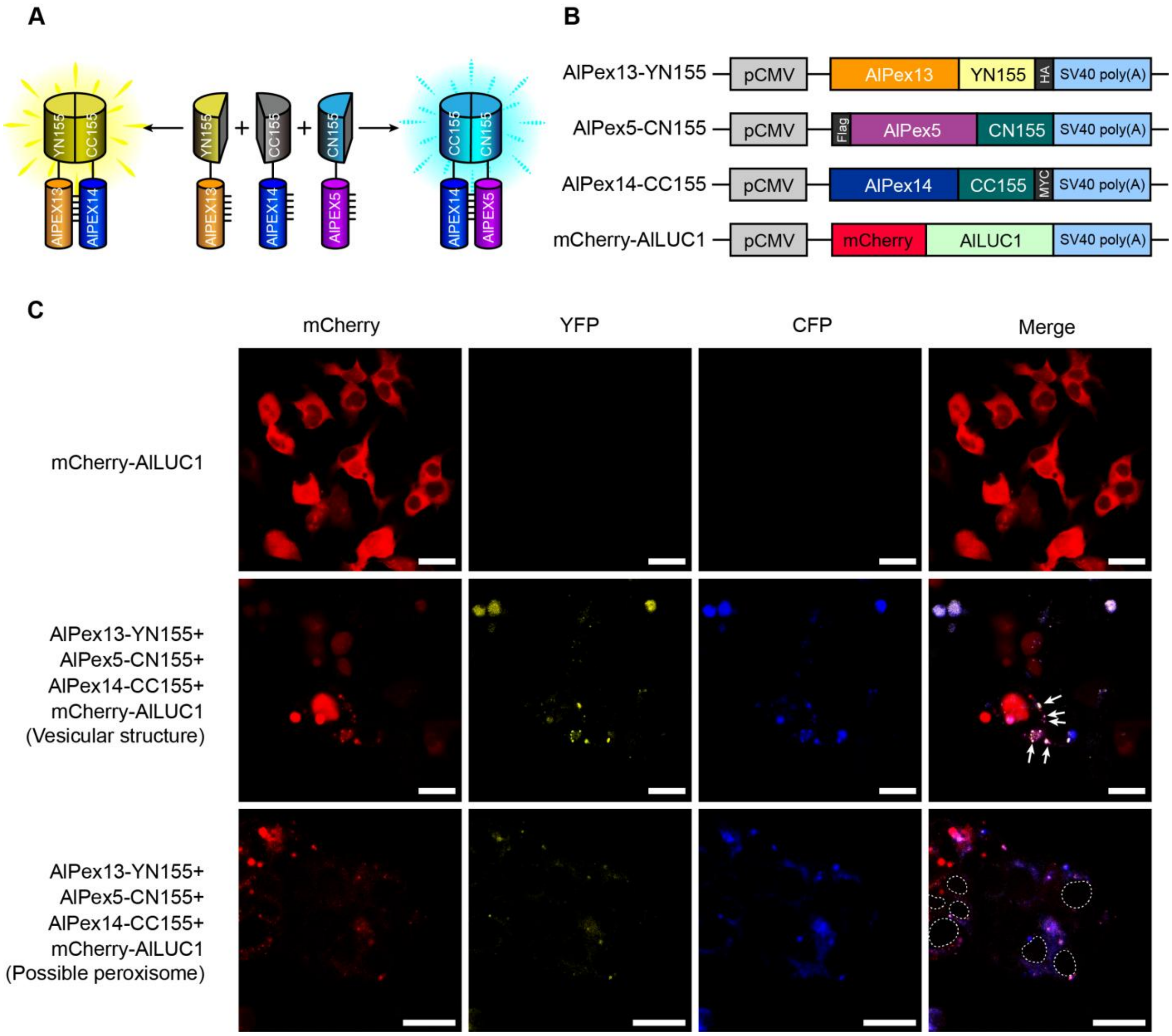

Supplementary Fig. 14. AlPEX13, AlPEX14, AlPEX5, AlPXMP2 and AlLUC1 interaction analysis by Multicolor BiFC in 293T cells. (A) Experimental basis of the multicolor assays. The AlPex13:AlPex14 heterodimer formation brings YN and CC together to form a yellow fluorescent protein (YFP), which could be detected at the YFP channel. The AlPex5:AlPex14 heterodimer formation brings CN and CC together to form an intact cyan fluorescent protein (CFP) protein, which could be detected using the CFP channel. **(B)** Vector constructs used in Multicolor BiFC. **(C)** Fluorescent images of co-expression of indicated plasmids. The addition of AlPEX13, AlPEX14 and AlPEX5 changed the subcellular localization of AlLUC1 (scale bar = 20 μm), n=4 independent experiments. The white arrowheads indicate colocalized signals. The white dashed areas indicate putative nuclei.

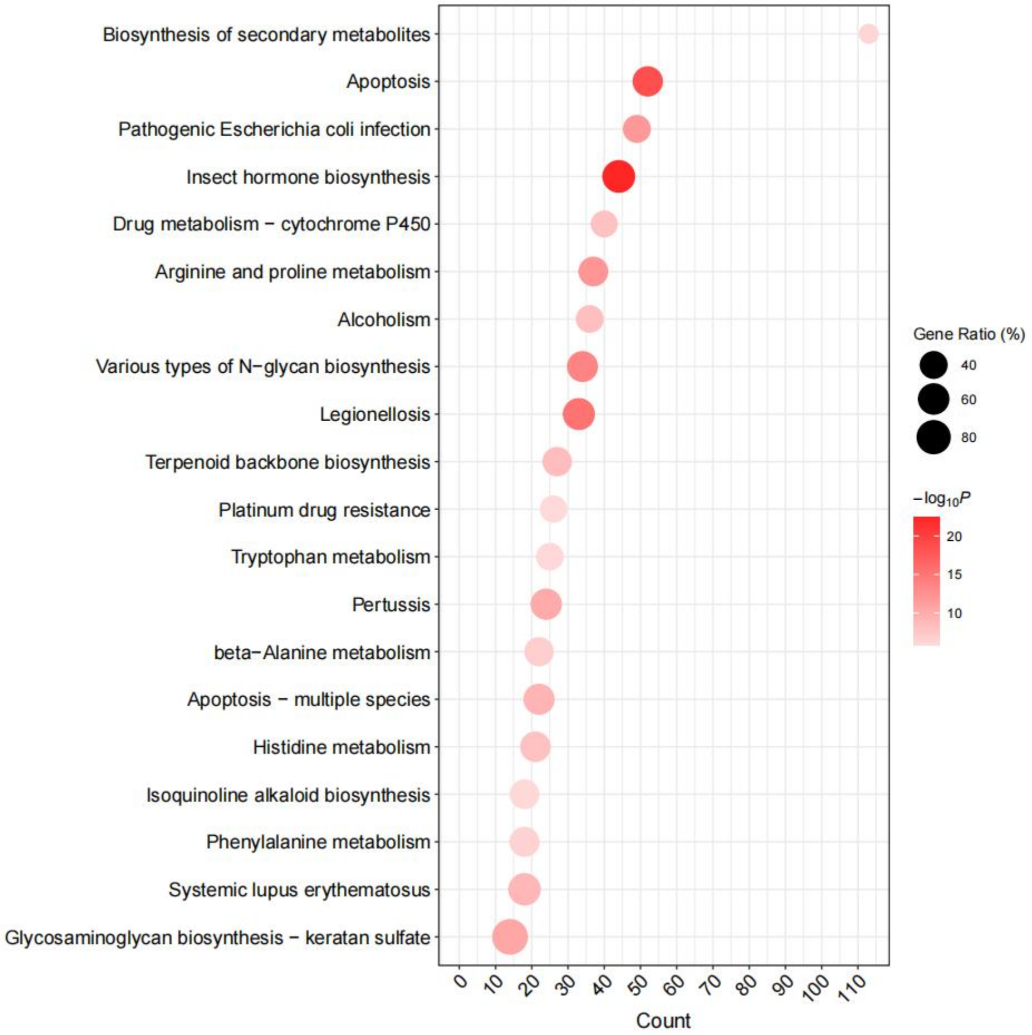

Supplementary Fig. 15. The KEGG enrichment of expanded gene families in *A. leii.* P value<E-06. Source data are provided as a Source Data file.

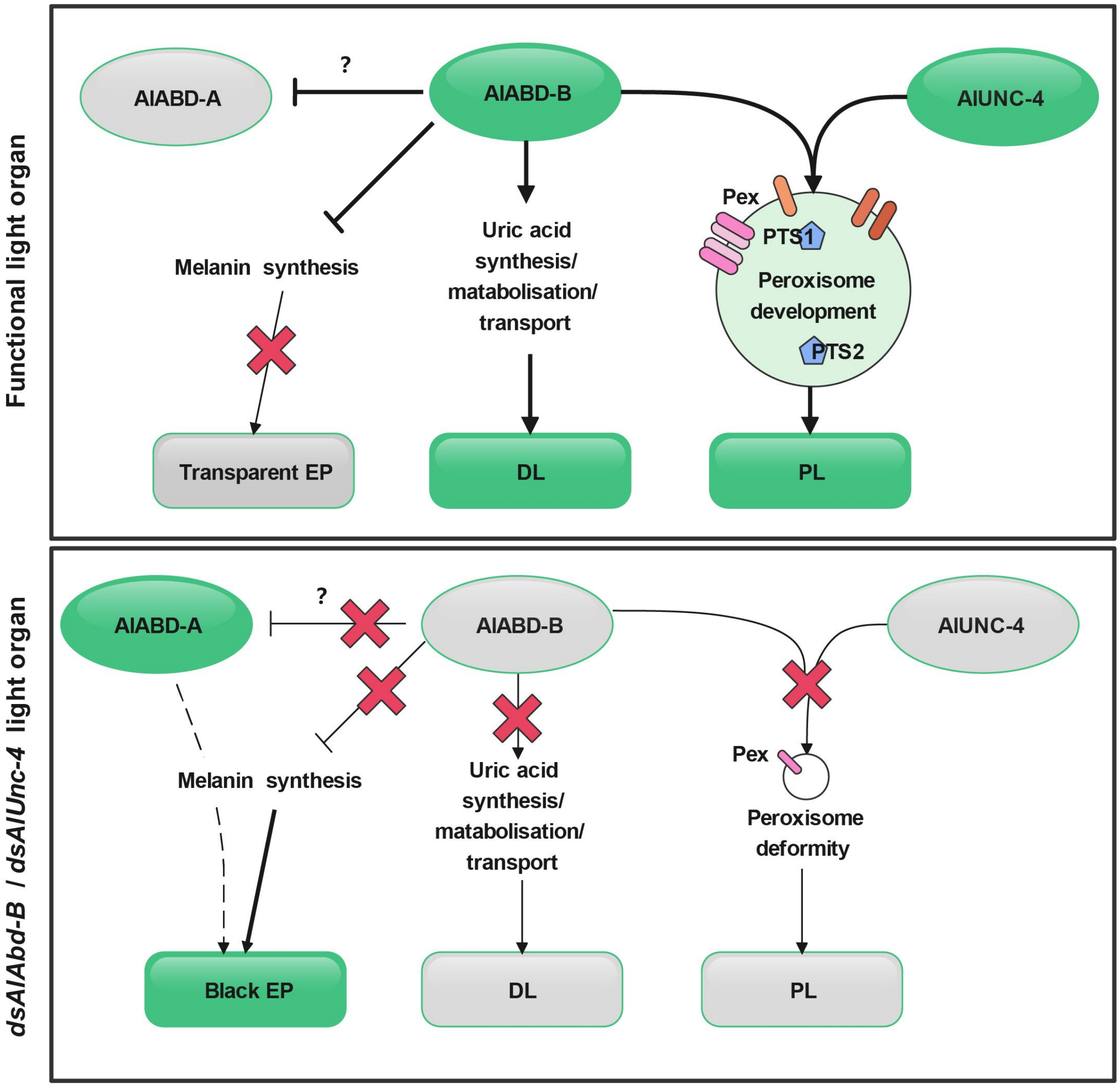

Supplementary Fig. 16. AlABD-B and AlUNC-4 are required for the development of the adult light organ and peroxisomes within it. During the development of the adult light organ in the pupal stage, the key transcription factor AlABD-B regulates the development of the light organ. AlABD-B may suppress the transcriptional expression and function of AlABD-A, suppress the synthesis of melanin, and cause transparency of the epidermis (EP) of light organs. AlABD-B also regulates the synthesis and transport of uric acid, which partakes in the development of the dorsal layer (DL). The development of peroxisomes in photocyte is also regulated by AlABD-B which partakes in the development of the photogenic layer (PL). A key transcription factor, AlUNC-4, participates in the development of PL. AlUNC-4 regulates the development of peroxisomes by interaction with AlABD-B. Decreased or absent expression of AlABD-B possibly results in the expression of AlABD-A, promotes the synthesis of melanin and darkens EP. A decrease in AlABD-B levels (or its absence) suppressed the synthesis and transport of uric acid and resulted in the destruction or absence of the entire dorsal layer. The absence of AlABD-B or AlUNC-4 resulted in deformed peroxisomes and non-luminescence. In the schematics, active genes are indicated in green coloring, inactive genes are indicated in gray coloring. bold lines indicate functional pathways, and thin lines indicate non-functional pathways. Lines terminating with an arrowhead indicate regulation in which the transcription factor functions as an activator, and lines terminating in a nail-head shape indicate repression. *AlAbd-B* or *AlUnc-4* RNAi lead to the pathway loss of function is indicated in red X.

Supplementary Tables

Supplementary Table 1. Statistics summary of filtered sequencing data for genome assembly.

Type	Platform	Total bases (Gb)	Depth (×)	Application
Third-generation	Nanopore	176.85	170	Assembly of initial contigs
Next generation	MGI2000	135.19	130	Genome survey and genomic base correction
HiC	Illumina NovaSeq	225.66	217	Chromosome construction

The sequencing depth was calculated by the assembled genome size 1.04G.

Supplementary Table 2. Hi-C scaffolding information of *A. leii* firefly.

Type	Contig Length	Contig Number	Scaffold Length	Scaffold Number	Gap Length	Gap Number
LG01	282,572,916	37				
LG02	197,929,339	45				
LG03	125,645,631	31				
LG04	115,159,544	10				
LG05	110,163,780	29				
LG06	88,271,171	18				
LG07	62,526,282	17				
LG08	51,297,175	4				
N50	10,806,843	27	125,648,631	3	100	92
N60	8,732,421	37	115,160,444	4	100	110
N70	6,305,189	52	110,166,580	5	100	129
N80	5,268,293	70	110,166,580	5	100	147
N90	2,773,805	97	62,527,882	7	100	165
Longest	40,465,452	1	282,576,516	1	100	183
Total	1,036,888,364	222	1,036,906,664	39	18300	183
Length>=1kb	1,036,888,364	222	1,036,906,664	39	0	0
Length>=2kb	1,036,888,364	222	1,036,906,664	39	0	0
Length>=5kb	1,036,888,364	222	1,036,906,664	39	0	0

Supplementary Table 3. Hi-C genome BUSCO forecast statistics of *A. leii* firefly.

Type	Number	Percent (%)
Complete BUSCOs (C)	1,342	98.2
Complete and single-copy BUSCOs (S)	1,331	97.4
Complete and duplicated BUSCOs (D)	11	0.8
Fragmented BUSCOs (F)	4	0.3
Missing BUSCOs (M)	21	1.5
Insecta	1,367	100

Supplementary Table 4. Comparisons of transposable elements (TEs) content in the assembled firefly genomes.

Species	Type	Length(bp)	% in genome
L. yunnana	DNA	367,551,516	34.91
	LINE	162,115,182	15.40
	SINE	2,204,348	0.21
	LTR	1,766,904	0.17
	Other	1,382	0.00
	Unknown	0	0.00
	Total	688,292,463	65.37
Abs. terminalis	DNA	88,325,208	17.62
	LINE	49,456,973	9.86
	SINE	715,335	0.14
	LTR	4,638,621	0.93
	Other	60	0.00
	Unknown	0	0.00
	Total	169,247,169	33.76
Pho. pyralis	DNA	118,270,813	25.08
	LINE	40,283,641	8.54
	SINE	1,748,154	0.37
	LTR	9,967,599	2.11
	Other	0	0.00
	Unknown	9,853,553	2.09
	Total	218,446,960	46.33
Pyr. pectoralis	DNA	96,920,898	18.21
	LINE	44,613,355	8.38
	SINE	1,069,705	0.20
	LTR	22,054,471	4.14
	Other	199,573	0.04
	Unknown	15,829,691	2.97
	Total	227,697,663	42.78
A. leii	DNA	218,672,799	21.09
	LINE	156,257,888	15.07
	SINE	2,204,339	0.21
	LTR	157,287,007	15.17
	Other	348,104	0.03
	Unknown	45,485,229	4.39
	Total	548,840,852	52.93

Supplementary Table 5. Gene comparison between *A. leii* genome and other reference genomes.

Gene set	Total number of protein coding genes	Average gene length (bp)	Average CDS length (bp)	Average number of exons per gene	Average exon length (bp)	Average intron length (bp)
A. leii	16,472	32,544	1478.74	5.62	262.98	6719.79
Pyr. pectoralis	13,292	14,140	1424.06	4.72	301.47	3414.77
Lam. yunnana	19,438	16,619	1377.84	4.74	290.54	3647.36
Abs. terminalis	20,438	7,767	1483.31	4.77	311.20	1532.06
Pho. pyralis	20,647	8,113	1489.2	5.15	289.09	1595.49
Agr. planipennis	13,371	11,946	1541.04	5.75	268.01	2190.54
Ano. glabripennis	14,828	16,649	1511.86	5.58	271.00	3305.94
I. luminosus	27,558	5373	1071.53	3.96	270.34	1266.76
Lep. decemlineata	14,000	14,162	1377.75	5.05	272.81	3156.44
O. taurus	14,537	7,834	1523.39	4.54	335.67	1783.58
T. castaneum	12,875	6,999	1550.97	5.28	293.75	1273.02
D. melanogaster	13,962	4,597	1560.59	3.90	400.29	1047.56

Supplementary Table 6. Functional annotation and completeness of proteins in the *A. leii* genome.

Type	Number	Percent (%)
Swissprot	10,879	66.05
KEGG	6,920	42.01
KOG	9,219	55.97
GO	8,336	50.61
NR	14,274	86.66
total Annotated	14,874	90.3
Gene	16,472	-
rRNA	156	
Small RNA	2023	
Regulatory(cis-regulatory elements)	678	
tRNA	667	

Supplementary Table 7. Reference genomes used in this study

Species	NCBI Submitted sequence Number
Drosophila melanogaster	GCA_000001215.4
Onthophagus taurus	GCA_000648695.2
Leptinotarsa decemlineata	GCA_000500325.2
Anoplophora glabripennis	GCA_000390285.2
Tribolium castaneum	GCA_000002335.3
Agrilus planipennis	GCA_000699045.2
Ignelater luminosus	GCA_011009095.1
Abscondita terminalis	GCA_013368085.1
Lamprigera yunnana	GCA_013368075.1
Pyrocoelia pectoralis	GCA_913698365.1
Photinus pyralis	GCA_008802855.1

Supplementary Table 8. Statistics of the numbers of transcription factor families in the *A. leii* genome

Super-family	Number of genes	Super-family	Number of genes
zf	590	P53	2
Homeobox	94	TSC22	2
bHLH	44	Tub	2
HTH	25	AF-4	1
HMG	24	CBF	1
MYB	20	CG-1	1
Fork head	18	COE	1
NF-YB	12	CSL	1
ETS	7	CSRNPN	1
ARID	6	DACH	1
T-box	6	GCM	1
MH1	5	LRRFIP	1
CSD	4	NDT80_PhoG	1
MBD	4	NF-YA	1
Runt	4	Nrf1	1
DM	3	PC4	1
E2F	3	SAND	1
HMGA	3	SRF	1
RFX	3	STAT	1
RHD	3	TEA	1
CP2	2	Others	7
HSF	2	Total	914
NF-YC	2		

Supplementary Table 9. Distribution of homeobox genes families.

Family	ANTP		PRD	LIM	POU	HNF	SINE	TALE	CUT	PROS	ZF	CERS	Other	Total
Class	HOXL	NKL												
Homo sapiens	52	67	98	12	24	3	6	30	10	2	15	5	9	333
Mus musculus	52	50	117	12	16	3	6	23	7	2	14	5	17	324
Gallus gallus	45	35	29	12	8	3	1	13	6	2	11	6	1	172
Danio rerio	68	64	53	20	19	6	13	29	9	3	17	3	18	322
Xenopus tropicalis	55	60	37	12	19	3	6	16	7	2	14	2	19	252
Branchiostoma floridae	24	36	29	7	7	4	3	9	4	1	5	1	3	133
Drosophila melanogaster	18	29	28	6	5	——	3	8	3	1	2	1	——	104
Tribolium castaneum	18	27	25	8	6	——	3	8	3	1	2	2	2	105
Apis mellifera	16	23	25	8	4	——	3	6	3	1	1	1	2	93
Caenorhabditis elegans	9	21	17	7	4	1	4	5	6	1	2	——	15	92
Aquatica leii	20	24	21	6	4	——	3	10	3	——	2	1	——	94

References

1 Vongsangnak, W., Chumnanpuen, P. & Sriboonlert, A. Transcriptome analysis reveals candidate genes involved in luciferin metabolism in *Luciola aquatilis* (Coleoptera: Lampyridae). *PeerJ* **4**, e2534 (2016).

2 Oba, Y., Yoshida, N., Kanie, S., Ojika, M. & Inouye, S. Biosynthesis of Firefly Luciferin in Adult Lantern: Decarboxylation of L-Cysteine is a Key Step for Benzothiazole Ring Formation in Firefly Luciferin Synthesis. *PloS ONE* **8**, e84023 (2013).

3 Hemmati, R. et al. Luciferin ‐ regenerating enzyme mediates firefly luciferase activation through direct effects of D ‐ cysteine on luciferase structure and activity. *Photochemistry and Photobiology* **91**, 828-836 (2015).

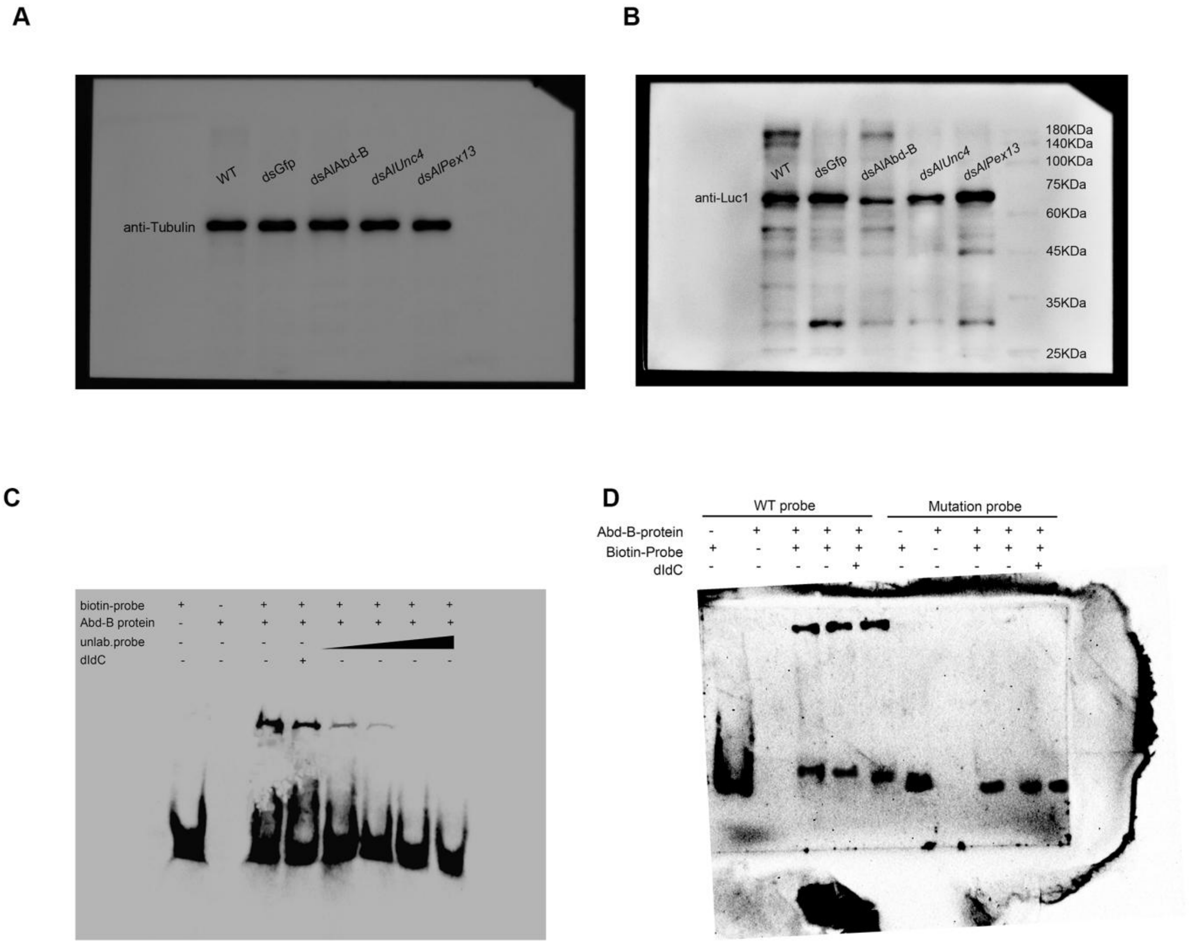

The uncropped scans of blots and gels. (A) and (B) Western blot for Supplementary Fig. 8B. (C) and (D) EMSA blot for Supplementary Fig. 11
