# Supplementary material for: Key homeobox transcription factors regulate the development of the firefly’s adult light organ and bioluminescence
Source: Nat Commun. 2024 Mar 5;15:1736. doi: 10.1038/s41467-024-45559-7 (PMC10914744; doi:10.1038/s41467-024-45559-7)
Supplement: Supplementary file 3 — Description of Additional Supplementary Files [file 41467_2024_45559_MOESM3_ESM.pdf]

Description of Additional Supplementary Files

File Name: Supplementary Data 1
Description: Statistics of the numbers of repetitive sequences of *A. leii* firefly.

File Name: Supplementary Data 2
Description: The list of homeobox genes in *A. leii* genome.

File Name: Supplementary Data 3
Description: Primers used in this study.

File Name: Supplementary Movie 1
Description: Phenotype of *dsAlAbd-A*, *dsAlAbd-B*, *dsAlAntp*, *dsAlUnc-4*, *dsAlRepo*, *dsAlAp2*, *dsAlUbx* and *dsAlShox2*.

File Name: Supplementary Movie 2
Description: Phenotype of *dsAlLuc1*.

File Name: Supplementary Movie 3
Description: Phenotype of *dsAlPex5*, *dsAlPex13*, *dsAlPex14* and *dsAlPxmp2*.
