# Supplementary material for: Key homeobox transcription factors regulate the development of the firefly’s adult light organ and bioluminescence
Source: Nat Commun. 2024 Mar 5;15:1736. doi: 10.1038/s41467-024-45559-7 (PMC10914744; doi:10.1038/s41467-024-45559-7)
Supplement: Supplementary file 10 — Reporting Summary [file 41467_2024_45559_MOESM10_ESM.pdf]

nature portfolio

Corresponding author(s): Xinhua Fu

Last updated by author(s): Jan 13, 2024

Reporting Summary

Nature Portfolio wishes to improve the reproducibility of the work that we publish. This form provides structure for consistency and transparency in reporting. For further information on Nature Portfolio policies, see our Editorial Policies and the Editorial Policy Checklist.

Statistics

For all statistical analyses, confirm that the following items are present in the figure legend, table legend, main text, or Methods section.

n/a	Confirmed	
☐	☒	The exact sample size (*n*) for each experimental group/condition, given as a discrete number and unit of measurement
☐	☒	A statement on whether measurements were taken from distinct samples or whether the same sample was measured repeatedly
☐	☒	The statistical test(s) used AND whether they are one- or two-sided<br>*Only common tests should be described solely by name; describe more complex techniques in the Methods section.*
☒	☐	A description of all covariates tested
☐	☒	A description of any assumptions or corrections, such as tests of normality and adjustment for multiple comparisons
☐	☒	A full description of the statistical parameters including central tendency (e.g. means) or other basic estimates (e.g. regression coefficient) AND variation (e.g. standard deviation) or associated estimates of uncertainty (e.g. confidence intervals)
☐	☒	For null hypothesis testing, the test statistic (e.g. *F*, *t*, *r*) with confidence intervals, effect sizes, degrees of freedom and *P* value noted<br>*Give P values as exact values whenever suitable.*
☒	☐	For Bayesian analysis, information on the choice of priors and Markov chain Monte Carlo settings
☒	☐	For hierarchical and complex designs, identification of the appropriate level for tests and full reporting of outcomes
☒	☐	Estimates of effect sizes (e.g. Cohen's *d*, Pearson's *r*), indicating how they were calculated

Our web collection on statistics for biologists contains articles on many of the points above.

Software and code

Policy information about availability of computer code

Data collection	Short-reads sequencing data were generated using MGISEQ2000 platform (GrandOmics, Wuhan, China); Long-reads sequencing data were generated using Nanopore PromethION sequencer (GrandOmics, Wuhan, China); Hi-C sequencing data were generated using Illumina Novaseq platform (GrandOmics, Wuhan, China); RNA concentration were measured using Nano-Drop 2000 spectrophotometer (Thermo Scientific); RNA-seq were generated using Illumina Hiseq platform (BGI, Shenzhen, China); Karyotype and Semi-thin sections were observed using Zeiss photomicroscope; qRT-PCR analysis was conducted using CFX Connect Real-time PCR Detection System (Bio-Rad, Hercules, CA); Ultrathin sections were observed using H7650 TEM microscope (Hitachi ,Tokyo, Japan); Flash rate and relative light intensity were recorded video camera (Sony A7s2); Luciferin were measured using 1260 Infinity II (Agilent); Gel and blot analysis was conducted using ChemiDoc™XRS + ((Bio-Rad, Hercules, CA); Immunofluorescence analysis were observed using Eclipse C1 laser-scanning microscope (Nikon, Tokyo, Japan); Subcellular and BiFC were conducted using SP8 lightning confocal microscope system (Leica, Heidelberg, Germany); Dual-luciferase reporter assay were conducted using EnVision multilabel plate reader (Perkin Elmer, Hopkinton, MA).
Data analysis	17-mer frequency distribution analysis was conducted using KMC (v3.2.1); The genome size was estimated using FindGSE56 and GenomeScope (v1.0.0); de novo genome assembly was conducted using NextDenovo (v2.3.0); Improvement of the accuracy was conducted using Racon v1.3.1 and Nextpolish (v1.2.4); The coverage of expressed genes was conducted using HISAT2 (v2.1.0); Clean paired-end reads mapped to the draft assembled sequence was conducted using bowtie2 (v2.3.2); Scaffolds clustered, ordered, and oriented onto chromosomes was conducted using LACHESIS (https://github.com/shendurelab/LACHESIS); Completeness and quality of the assembly were checked using BUSCO (v5.1.3) and insecta_odb10 database; Raw sequences were filtered using Trimmomatic (v0.39); RNA clean reads mapped using HISAT2 (v2.1.0); Gene expression level was conducted using Stringtie (v1.3.4); DEGs were identified using DESeq2 (v1.40.2); Tandem repeats were identified using GMATA (v2.2) and TRF (v4.07b); ab initio prediction was conducted using AUGUSTUS (v3.3.1); Homology search was conducted using GeMoMa (v1.6.1); Reference-guided transcriptome assembly was conducted using PASA(v2.3.3); Functional annotation was conducted using Blastp (v2.7.1); tRNAs were predicted using tRNAscan-SE (v2.0); MicroRNA, rRNA, small nuclear

RNA, and small nucleolar RNA were scanned using Infernal (v1.1.2); rRNAs were predicted using RNAmmer (v1.2); Orthology analysis was conducted using OrthMCL(v2.5.4); Gene enrichment was conducted using Gene Ontology (http://www.geneontology.org/) KEGG (Kyoto Encyclopedia of Genes and Genomes, https://www.kegg.jp/kegg/); Orthologs was aligned using MUSCLE (v3.8.31); Alignments were processed using Gblocks (v0.91b); Best model for phylogenetic trees was selected using ProtTest (v3.0); Phylogenetic trees were generated PhyML (v3.0); Tree file was visualized using Figtree (v1.4.3); Species divergence time was estimated using RelTime of MEGA-CC (v10.1.8); Identification of transcription factors was conducted using AnimalTFDB (v3.0); Identification of the homeobox gene family was conducted using HMMER (v3.0) and Blastp (v2.7.1); Structural predictions was conducted using SMART (v9.0); Homeobox protein sequences were aligned using MAFFT (v7.5); Gap sites remove was conducted using trimAl (v1.2); The best-fit substitution model was selected using ModelFinder (part of IQ-TREE version 1.6.1); Phylogenetic tree construct was conducted using IQ-TREE (v2.1.2); cDNA cloning and protein sequence were predicted using DNAMAN (v7.0.2); PST1 signal peptides were predicted using PSORT Prediction (http://psort1.hgc.jp/form.html); Phylogenetic trees of AlLuc1 and AlLuc2 were built using MEGA7 (7.0.21); Primers were designed using Primer3 web tool (https://primer3.ut.ee/); Luciferin retention time and peak area were measued using OpenLab CDS2 (Agilent); Statistical analyses were conducted using Origin (v8.5.0).

For manuscripts utilizing custom algorithms or software that are central to the research but not yet described in published literature, software must be made available to editors and reviewers. We strongly encourage code deposition in a community repository (e.g. GitHub). See the Nature Portfolio guidelines for submitting code & software for further information.

Data

Policy information about availability of data

All manuscripts must include a data availability statement. This statement should provide the following information, where applicable:

- Accession codes, unique identifiers, or web links for publicly available datasets
- A description of any restrictions on data availability
- For clinical datasets or third party data, please ensure that the statement adheres to our policy

Source data are provided with this paper. The genome assemblies and sequence data A. leii were deposited in NCBI under the BioProject accession number PRJNA948550 (https://www.ncbi.nlm.nih.gov/bioproject/PRJNA948550), the whole genome project has been deposited at DDBJ/ENA/GenBank under the accession JARPUR010000000 (https://www.ncbi.nlm.nih.gov/nuccore/JARPUR000000000.1), and CNCB-NGDC (National Genomics Data Center, China National Center for Bioinformation) under the BioProject accession number PRJCA016073 (https://ngdc.cncb.ac.cn/bioproject/browse/PRJCA016073).

Research involving human participants, their data, or biological material

Policy information about studies with human participants or human data. See also policy information about sex, gender (identity/presentation), and sexual orientation and race, ethnicity and racism.

Reporting on sex and gender	not applicable
Reporting on race, ethnicity, or other socially relevant groupings	not applicable
Population characteristics	not applicable
Recruitment	not applicable
Ethics oversight	not applicable

Note that full information on the approval of the study protocol must also be provided in the manuscript.

Field-specific reporting

Please select the one below that is the best fit for your research. If you are not sure, read the appropriate sections before making your selection.

☒ Life sciences ☐ Behavioural & social sciences ☐ Ecological, evolutionary & environmental sciences

For a reference copy of the document with all sections, see nature.com/documents/nr-reporting-summary-flat.pdf

Life sciences study design

All studies must disclose on these points even when the disclosure is negative.

Sample size	In this study, The same 1-day-old adult (after emergence from the pupae stage) male specimen was used for both MGI and ONT genome sequencing. Another 1-day-old adult male specimen was used for Hi-C sequencing. for RNA-seq and functional experiments, more than five individuals was chosen for each replicate, not less than three replicates were used in this study, which are generally sufficient for statistical test. The precise sample sizes have been provided in the Methods section and figure legends.
Data exclusions	No data were excluded from the analyses.
Replication	All the data were collected by 3 individual experiments. All attempts at replication were successful.

Randomization	In this study, The RNA-seq were performed based on different developmental stages of A.leii. For functional study on genes, 1-d-puape randomly used for RNAi. For data analysis, more than 3 replicates at each group were chosen randomly.
Blinding	All the experiments were performed by the investigators that were blinded to group allocations

Reporting for specific materials, systems and methods

We require information from authors about some types of materials, experimental systems and methods used in many studies. Here, indicate whether each material, system or method listed is relevant to your study. If you are not sure if a list item applies to your research, read the appropriate section before selecting a response.

Materials & experimental systems

n/a	Involved in the study
☐	☒ Antibodies
☐	☒ Eukaryotic cell lines
☒	☐ Palaeontology and archaeology
☐	☒ Animals and other organisms
☒	☐ Clinical data
☒	☐ Dual use research of concern
☒	☐ Plants

Methods

n/a	Involved in the study
☒	☐ ChIP-seq
☒	☐ Flow cytometry
☒	☐ MRI-based neuroimaging

Antibodies

Antibodies used	rabbit antibody to Firefly Luciferase (Bioss, bs-8539R), mouse monoclonal antibody to α-tubulin (Beyotime, AT819), goat-anti-Rabbit IgG-HRP(Beyotime, A0208), goat anti-mouse IgG-HRP (Beyotime, A0216), rabbit anti-Firefly Luciferase primary antibodies conjugated with Alexa Fluor 594 (Bioss, bs-8539R-AF594)
Validation	We selected these antibodies considering their wide use by the research community. In addition, they have been validated by the manufacturer. The detail information of each antibody with references is given below:<br>http://www.bioss.com.cn/prolook_03_biaoji.asp?pro2a=20120816081636194652&pro33=221<br>http://www.bioss.com.cn/prolook_03_biaoji.asp?pro2a=20120816081636194652&pro33=221<br>https://www.beyotime.com/Manual/AT819%20Tubulin%E6%8A%97%E4%BD%93.pdf

Eukaryotic cell lines

Policy information about cell lines and Sex and Gender in Research

Cell line source(s)	HEK293T cells (pricella, CL-0005)
Authentication	HEK293T cell line was authenticated by Procell life science&technology Co.,Ltd. when we purchased them, Procell uses STR analysis to authemticate their cell lines. we did not independently authenticate them.
Mycoplasma contamination	All cell lines were confirmed mycoplasma negative. we conduct routine mycoplasma testing in the laboratory every month.
Commonly misidentified lines (See ICLAC register)	none of the commonly misidentified cell lines were employed in this study

Animals and other research organisms

Policy information about studies involving animals; ARRIVE guidelines recommended for reporting animal research, and Sex and Gender in Research

Laboratory animals	Larvae, pupae and adults were obtained from the aquatic firefly Aquatica leii breeding lab (a lab established in Wuhan City, Hubei Province, solely to breed A. leii from the original firefly population collected from Hangzhou City, Zhejiang Province), and kept in the laboratory at 25 ± 1°C for 24 h under 70 ± 5% humidity and a 14:10 h light/dark (L:D) photoperiod. The same 1-day-old adult (after emergence from the pupae stage) male specimen was used for both MGI and ONT genome sequencing. Another 1-day-old adult male specimen was used for Hi-C sequencing. Twenty 5th-instar larvae were used for karyotype observation. Tissues corresponding to the adult light organs (ventrites 6 and 7, abdomen cuticle) were dissected from male pupae specimens at different developmental stages (1d-pupae, 3d-pupae, and 5d-pupae) for RNA-seq. Both male and female one-day-old pupae were injected with 2 µg of dsRNA (total injection volume 500 nL per insect) through the abdominal segment membrane, between V6 and V7.
Wild animals	No wild animals were used or captured in this study.
Reporting on sex	Both sexes were used in this study.

Field-collected samples	No field-collected samples were used in this study.
Ethics oversight	The animal experiments were performed according to the procedures approved by the Laboratory Animal Welfare and Ethics Committee of Huazhong Agricultural University.

Note that full information on the approval of the study protocol must also be provided in the manuscript.

Plants

Seed stocks	*Report on the source of all seed stocks or other plant material used. If applicable, state the seed stock centre and catalogue number. If plant specimens were collected from the field, describe the collection location, date and sampling procedures.*
Novel plant genotypes	*Describe the methods by which all novel plant genotypes were produced. This includes those generated by transgenic approaches, gene editing, chemical/radiation-based mutagenesis and hybridization. For transgenic lines, describe the transformation method, the number of independent lines analyzed and the generation upon which experiments were performed. For gene-edited lines, describe the editor used, the endogenous sequence targeted for editing, the targeting guide RNA sequence (if applicable) and how the editor was applied.*
Authentication	*Describe any authentication procedures for each seed stock used or novel genotype generated. Describe any experiments used to assess the effect of a mutation and, where applicable, how potential secondary effects (e.g. second site T-DNA insertions, mosiacism, off-target gene editing) were examined.*
